# Duties of Anaesthetists and Assessment of Awareness, Concerns, and Expectations on Anaesthesia Practices

**DOI:** 10.4274/TJAR.2023.231328

**Published:** 2023-12-27

**Authors:** Melahat Yalçın Solak, Murat İzgi, Murat Tümer, Şennur Uzun

**Affiliations:** 1 Clinic of Anaesthesiology and Reanimation, Hacettepe University Hospital, Ankara, Turkey; 2 Clinic of Anaesthesiology and Reanimation, American Hospital, İstanbul, Turkey

**Keywords:** Anaesthesia, anxiety, knowledge

## Abstract

**Objective:**

Numerous studies performed worldwide indicate that the public has limited knowledge of anaesthesia practices and anaesthetists’ duties and responsibilities. This study aimed to identify the level of knowledge about anaesthetists and anaesthesia practices, and to assess the reasons for anxiety about anaesthesia of the population admitted to our hospital, which is tertiary in Turkey. The secondary aim was to analyze their differences according to sex, education level, and acquired anaesthesia experience.

**Methods:**

A survey comprising 23 questions was administered to 400 patients and/or their relatives, aged 18-85 years, who presented to our clinic for preoperative anaesthesia evaluation and for whom elective surgery was planned from March through October 2017.

**Results:**

Of the 400 participants, 213 were women and 187 were men. Of all participants in the survey, 51.2% were patients and 48.8% were patient relatives; 64.2% had anaesthesia experience and 35.8% had never had anaesthesia before. The survey group’s level of knowledge about anaesthesia was generally low. According to education level, there was a statistically significant difference in the anaesthesia recognition level. However, the acquired anaesthesia experience did not affect the anaesthesia recognition level.

**Conclusion:**

To raise the level of knowledge about this topic, anaesthetists must provide patients with more detailed information during preoperative and postoperative visits, which would significantly reduce their anxiety levels. Further, we determined that increasing the use of methods such as media-based brochures, booklets, and videos to inform patients may increase knowledge levels and reduce anxiety levels.

**Main Points**

• Numerous studies performed worldwide indicate that the public has limited knowledge of anaesthesia practices and anaesthetists’ duties and responsibilities. In this study, we aimed at our hospital population’s knowledge and anxiety levels.

• The survey group’s level of knowledge was generally low. According to education level, there was a statistically significant difference in the anaesthesia recognition level. However, the acquired anaesthesia experience didn’t affect it.

• We determined that increasing preoperative and postoperative visits to inform patients and using media-based items may have a role in increasing patients’ knowledge levels and reducing their anxiety levels.

## Introduction

Developments in the area of anaesthesia contribute to successful surgical outcomes at a growing rate.^1^ However, the roles played by anaesthetists in the operating room (OR) and the responsibilities that they share with the surgeon are not known.^2^ Although anaesthetists have several duties such as resuscitation, intensive care, and acute and chronic pain management, in addition to their duties in the OR, several studies performed across the world show that society has limited knowledge about anaesthesia practices and anaesthetists’ duties and responsibilities. The level of knowledge about this topic varies according to certain factors such as socioeconomic status, education level, age, sex, and anaesthesia experience.^3,4,5,6,7,8,9,10^ The patient’s limited level of knowledge about anaesthesia increases anxiety, which adversely affects the perioperative period and postoperative recovery.^9,11^

Through a survey administered to the patients and/or their relatives who were admitted to our hospital for elective surgery, we aimed to evaluate a part of Turkish society’s level of knowledge about anaesthesia and anaesthetists, to determine the reasons for anxiety, and to compare the differences in knowledge and anxiety according to sex, education level, and acquired anaesthesia experience, to identify inaccurate or missing knowledge about the topic, and determine what anaesthetists should do about this topic.

## Methods

Ethical approval for this study (approval no: GO 17/113-27) was provided by the Non-Invasive Clinical Research Local Ethics Committee of Hacettepe University, Ankara, Turkey on February 28^th^, 2017.

The research participants were patients and/or their relatives, aged 18-85 years, who were admitted to the anaesthesia clinic in Hacettepe University Hospital between March and October 2017 for preoperative anaesthesia assessments, agreed to participate in the survey, and knew Turkish.

Patients and/or patient relatives who had a brain injury, speech and hearing disorders, a previously diagnosed serious psychiatric problem, or a serious illness that would affect the general health condition were excluded from the study. A total of 400 patients and/or patient relatives were included in the study.

The survey form contained 23 questions, which were presented in two parts. The first part of the survey addressed demographic data such as age, sex, and education level, and the second part contained questions about anaesthetists’ duties, workplaces, anaesthesia methods, and reasons for having anaesthesia-related fear and anxiety.

After the patients and/or their relatives consented to participate in the study upon being informed about the survey by the anaesthetist, they were asked to complete the survey form. The survey was administered verbally to participants and/or patient relatives who were illiterate and their verbal responses were recorded.

### Statistical Analysis

Statistical analysis of the research data was conducted using the Statistical Package for the Social Sciences (SPSS) ver. 20.0 software package. Whether the research data were normally distributed was checked using the Shapiro-Wilk test. Numerical variables that were normally distributed are expressed as “mean ± standard deviation”. Categorical variables are presented as numbers and percentages. In the case of numerical variables with a normal distribution, the independent samples t-test was used to compare the two groups. The chi-square test, Yates’s correction for continuity, and Fisher’s exact test were used to compare categorical data. Statistical significance was identified if the *P* value was lower than 0.05 (*P* < 0.05).

## Results

The study population comprised 400 patients/relatives. Two hundred thirteen were female (53.3%) and 187 were male (46.7%). The demographic data of the research participants are presented in Table 1.

Of our study population, 51.2% were patients and 48.8% were patient relatives, 68% had anaesthesia experience, and 32% had never had anaesthesia before. Three-quarters (76.5%) of the participants answered the question “Who do you think performs the anaesthesia in surgery?” as an anaesthesiologist, 12.5% answered it as “I do not know.” The majority of the participants (69%) answered the question “Who is the anaesthesiologist?” as “A specialist physician who graduated from medical school and received anaesthesia training;” the rate of those who answered saying “I don’t know” was 24.5%. Of all the participant patients/relatives, 85% knew about general anaesthesia, 63.8% knew about local anaesthesia, and 61% knew about regional anaesthesia from among the anaesthesia methods. Female participants had a higher percentage of knowledge of regional anaesthesia than male participants and this difference was statistically significant (67.6% vs. 53.5%, *P*=0.004). For the question about anaesthetist’s duties in the perioperative process, 77.3% of the participants said “Anaesthetist follows up the patient’s sleep and wakefulness, that is, the patient’s state of consciousness.” In the framework of the question about the anaesthetist’s duties outside the operation room, 36.5% of the participants said, “Anaesthetists work in intensive care units (ICUs) called reanimation,” and only 29.5% of the participants responded, “Anaesthetists take part in the treatment of a variety of pains in particular cancer pain.” The answers to these questions did not differ significantly in terms of sex (Table 2).

Compared with the women, the ratio of getting information about anaesthesia from “internet-press” (22.6% vs. 34.2%; *P*=0.010), the ratio of getting information from “surgeon” (18.3% vs. 28.3%; *P*=0.017) and the ratio of obtaining information from “friends-neighbours” (20.7% vs. 29.9%; *P*=0.032) were found to be significantly higher for the male participants. For the answers of the participants to the question, “By whom would you like to be informed before surgery about anaesthesia?”, no significant difference was found in terms of sex (Table 3).

In comparison with other groups of participant patients/patient relatives with different education levels, the group of participants with college/university degrees had higher levels of knowledge about anaesthesia practices, anaesthetists’ duties in the perioperative process, and their areas of work outside the OR, and this difference was statistically significant (*P *< 0.001). It was ascertained that, as the education level increased, the percentage of participants who acquired knowledge about anaesthesia from the internet/media (*P *< 0.001) and spouse/friends/neighbours (*P*=0.003) also increased.

It was found that, as per the education level, the participants with college/university degrees had a higher percentage of fear of anaesthesia than other groups of participants, and this difference was statistically significant (*P*=0.048). On the other hand, as per the education level, there was no statistically significant difference between the participants regarding the reasons for fearing anaesthesia (*P *> 0.05).

Based on previous surgeries under anaesthesia, there was no statistically significant difference in the participants’ levels of knowledge about anaesthesia practices, anaesthetists’ duties in the perioperative process, and their areas of work outside the OR (*P *> 0.05). In addition, the participants who had previously been under anaesthesia were also compared in terms of the scale of the surgical procedure. However, there was no statistically significant difference in their knowledge about anaesthesia, anaesthetists, and anxiety about anaesthesia (*P* > 0.05).

The ratio of patients/patient relatives who stated that they were afraid of anaesthesia was 53.3%, and a higher rate was observed in women than in men (58.7% vs. 47.1%; *P*=0.020). One-fifth (21.3%) of the participants were afraid of waking up during surgery, 22.3% were of feeling pain during surgery, 17.5% were afraid of nausea-vomiting, 14.5% were afraid of losing consciousness and saying something they did not want to, 8% were afraid of having a sore throat, 34.5% were scared about not being able to wake up from anaesthesia, and 13.5% were afraid of death. Nausea-vomiting (22.1% vs. 12.3%; *P*=0.010) and fear of not waking up from anaesthesia (42.3% vs. 25.7%; *P *< 0.001) were found to be higher in women compared with men (Table 4).

**Figure 1:**
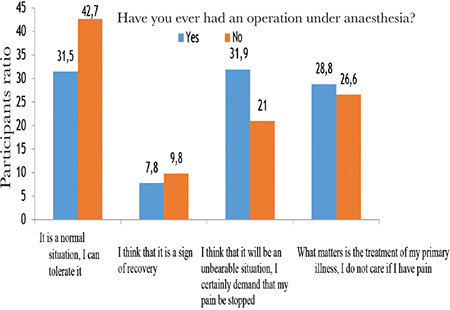
Percentage of the participants’ views about postoperative pain based on having undergone surgery previously

For the question, “What do you think about post-surgery pain?”, 35.5% of the patients/patient relatives answered “It is a normal situation, I can tolerate it”, 8.5% said “I think it is a sign of recovery”, 28% responded “I think it will be an unbearable situation, I would definitely like my pain to stop”, and 28% as “The important thing is the treatment of my primary disease, I don’t care if I have pain or not.” The ratio of participants who answered, “It is a normal situation, I can tolerate it” was higher in men, and the rate of those who answered “I think it will be an unbearable situation, I would definitely like my pain to stop” was higher in women. In addition, the ratio of those who wanted postoperative pain to stop was found to be significantly higher for patients who had undergone surgery previously (Figure 1).

## Discussion

Along with the development of technology, anaesthesia practice has made considerable progress in recent years.^1^ Despite these developments, society has limited knowledge about anaesthesia practices, anaesthetists’ duties, and responsibilities according to several studies performed worldwide.^1,5,12^

The results of studies conducted to determine whether anaesthetists were specialists varied across countries. For instance, 51.75% of patients in Brazil,^13^ 55.3% of the patients in Saudi Arabia,^5^ 56% of patients in Pakistan,^12^ 59% of patients in Latin America,^4^ 74.8% of patients in South Korea,^14^ 86% of patients in the state Minnesota in the United States of America (USA),^15^ 92.3% of patients in Israel^7^, and 99% of patients in Switzerland^16^ knew that the anaesthetist was a specialist physician. According to the results of these studies, it can be asserted that, in line with the development level of countries, there is an increase in the percentage of knowledge of the anaesthetist. In the present study, 69% of the participants stated that the anaesthetist was a specialist physician with education in anaesthesia. In comparison with the results of studies performed across the world, it can be said that this percentage is about average. Nevertheless, the anaesthetist, in whom patients entrust their lives, was still not known by one-third of the participants of our study. The reasons for the inadequacy of knowledge about anaesthetists may be that the patients present to a surgeon first, they are forwarded by the surgeon to the anaesthetist, and their meetings with the anaesthetist were short relative to their encounters with other specialist physicians.

Patients experience intense anxiety due to anaesthesia and subsequent surgery. In addition, they do not know exactly the division of labor in the OR and who is responsible for each practice. A study conducted by sharing the patient data of Australia, Germany, and the USA showed that patients were partially informed about the perioperative process.^6^ In our study, about the question about the anaesthetist’s duties in the perioperative process, 77.3% of the participants said, “Anaesthetist follows up the patient’s sleep and wakefulness, that is, patient’s state of consciousness,” 48.3% of the participants responded, “Anaesthetist follows up the patient’s blood pressure, pulse, respiration, and other vital signs,” 52.8% of the participants stated, “Anaesthetist ensures that the patient feels no pain,” and 33.8% of the participants reported, “Anaesthetists identify which serum, blood, and blood products are necessary for the patient and assures that serum and/or blood and/or blood products are given to the patient at the quantity that they deem adequate.” As per these data, it was discerned that the participants of the current study had inadequate knowledge about the perioperative resuscitative process.

Outside the OR, the anaesthetist gradually becomes more involved with duties such as resuscitation, intensive care, acute and chronic pain management, and radiology examinations and interventions. In several countries, ICUs are managed by anaesthetists. Some 17% of patients in Minnesota in the USA,^15^ 57% of patients in Finland,^17^ 20.2% of patients in South Korea,^14^ and 46% of the patients in Israel^7^ knew that the anaesthetist worked in ICUs, and 20% of patients in Minnesota in the USA,^15^ 22% of patients in Finland,^17^ 28.7% of patients in South Korea,^14^ and 33.8% of patients in Israel^7^ knew that anaesthetists worked in pain centers. It was found that, of the participants in the current study, 36.5% knew that anaesthetists worked in ICUs, 61.7% knew that anaesthetists worked in preoperative assessment clinics, 29.5% knew that anaesthetists worked in pain centers, and 37.5% knew that anaesthetists worked in places such as endoscopy units, nephrolithotomy, cardiology, and angiography laboratories, and radiology monitoring centers. A comparison of the results of the current study and those of studies conducted in other countries, shows that the results of our study are around the average values and the knowledge about this topic in our society and across the world is inadequate. The reasons for the inadequacy of knowledge about this topic could be that anaesthetists have acquired a professional identity only recently, anaesthetist’s duties have expanded at an ever-increasing rate in recent years, and anaesthetists work as consultant physicians.

Being informed is accepted as an important patient right today.^18^ In addition to preoperative evaluation, informed consent forms are used in our hospital to inform patients about anaesthesia. In our study, when we questioned where the participants attained their knowledge about anaesthesia, the ratio of those who learned it from an “anaesthesiologist” was highest at 50.8%, followed by “internet-press” (28.1%). An increase was observed in the ratio of those who learned their knowledge from “internet-press” in proportion to the increase in education level. While giving information, in addition to preoperative evaluations, media-based brochures, booklets, videos, and audio recordings can be used, and it has been shown that the use of these materials increases the knowledge level of patients and reduces their level of anxiety.^19^ We think that it may be beneficial to use various communication tools to raise awareness on this issue.

Education level is one of the parameters used frequently in research to compare people’s levels of knowledge about anaesthetists’ duties and anaesthesia practices. A study by Eyelade et al.^18^ stated that patients with degrees from tertiary education institutions had higher levels of knowledge about anaesthesia and anaesthetists. Shevde and Panagopoulos^19^ found that there was no relationship between education level and knowledge about anaesthesia. In the current study, it was ascertained that participants with college/university degrees had higher levels of knowledge about anaesthesia, anaesthetists, and anaesthetists’ duties inside and outside the OR, and this difference was statistically significant. This situation can be connected to the fact that participants with relatively high education levels were more curious about anaesthesia and surgery, made more effort, and used tools such as the internet more frequently to reach the knowledge that they aspired to obtain.

In a study conducted in Israel by Calman et al.,^1^ the patients were categorized as those who had anaesthesia before and those who would be anaesthetized for the first time. Subsequently, the patient’s levels of knowledge about anaesthesia practices and the anaesthetist were evaluated, and it was found that past experiences did not affect the patient’s knowledge levels. Baja et al.^20^ identified that the level of knowledge about anaesthesia increased along with past experiences, and the patients who had anaesthesia before had higher levels of knowledge than those who were curious about anaesthesia. However, in the current study, it was found that experience did not affect the level of knowledge about anaesthesia. Our study also investigated whether the severity of the surgeries that participants had previously undergone affected patients’ levels of knowledge about anaesthesia and anaesthesiologists. However, this difference was not statistically significant.

It is common for patients to be afraid of anaesthesia in the preoperative period, and this situation adversely affects their surgery and postoperative recovery.^11^ While more than half (53.3%) of the participants in the current study reported that they had a fear of anaesthesia, it was discerned that a higher percentage of female participants had a fear of anaesthesia than the male participants. Several studies have identified that females had a higher rate of anxiety than males. This situation may stem from males’ desire to look powerful in the context of the existing sociocultural structure. Shevde and Panagopoulos^19^ proposed that different results could be obtained if the test was performed by a psychologist.

According to previous research, in general, patients experience varying degrees of anxiety about situations such as being unable to wake up even if the anaesthetic is stopped, death, brain injury, paralysis, anaesthesia awareness, feeling pain during surgery, making meaningless comments while anaesthetized, feeling nauseous, vomiting, inadequate knowledge and experience of the anaesthetist, and the absence of an anaesthetist in the OR. According to the study by Nagrampa et al.,^21^ patients mostly feared pain, followed by the fear of dying and having a brain injury. According to the studies by Ribeiro and Mourão^13^ and Gottschalk et al.,^6^ the patients’ greatest fears were being unable to wake up after the surgery and having a postoperative infection. According to the study by Shevde and Panagopoulos^19^, patients’ fear was the anaesthetist’s professional inadequacy. Matthey et al.^22^ found that patients in their study feared awareness during surgery. In our study, the patients’ greatest fear was that they would be unable to wake up even if the anaesthetic was stopped.

According to whether the participants had undergone surgery with anaesthesia before and the severity of the surgery, no significant difference was observed among them between the fear rates of those who feared anaesthesia and the reasons for their fear. When the fears of our participants about anaesthesia were examined according to their education level, the rate of those who were afraid of anaesthesia was found to be higher in the college/university education group compared with the other education groups. However, there was no difference according to education level in terms of fear reasons. Observing a higher level of anxiety in patients with a high educational level can be attributed to having more information about complications.

In light of the responses to the questions about postoperative pain, it was considered that the female participants were more anxious about postoperative pain than the male participants. Patients who had undergone surgery before and had anaesthesia experience, want their pain to stop.

### Study Limitations

The limitations of our study are the inability to generalize about the level of social knowledge because it is a cross-sectional and single-center study, and the inability to include the thoughts and comments of the patients that would contribute to the study, beyond the fixed questions and answers, because it is a questionnaire study.

## Conclusion

In this survey, we evaluated the level of knowledge and concerns about anaesthesia applications and anaesthesiologists among our research participants. We found that the level of knowledge on this subject was insufficient and this lack of knowledge increased the level of anxiety in the preoperative period. We think that more objective results can be obtained on the level of knowledge and concern of our society by conducting national-based multi-centered studies. In light of this information, we concluded that, in addition to evaluating the patients in terms of the surgery, giving detailed information about the conditions and practices that would be experienced during the procedure and answering questions of the patients during preoperative visits would significantly reduce their anxiety levels. We determined that increasing the use of methods such as media-based brochures, booklets, videos, audio tapes, and preoperative and postoperative visits to inform patients may have a role in increasing patients’ knowledge levels, reducing their anxiety levels, and increasing patient satisfaction.

## Figures and Tables

**Table 1:**
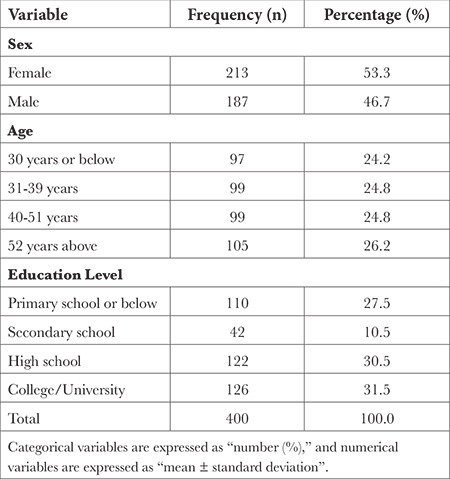
Demographic Characteristics

**Table 2:**
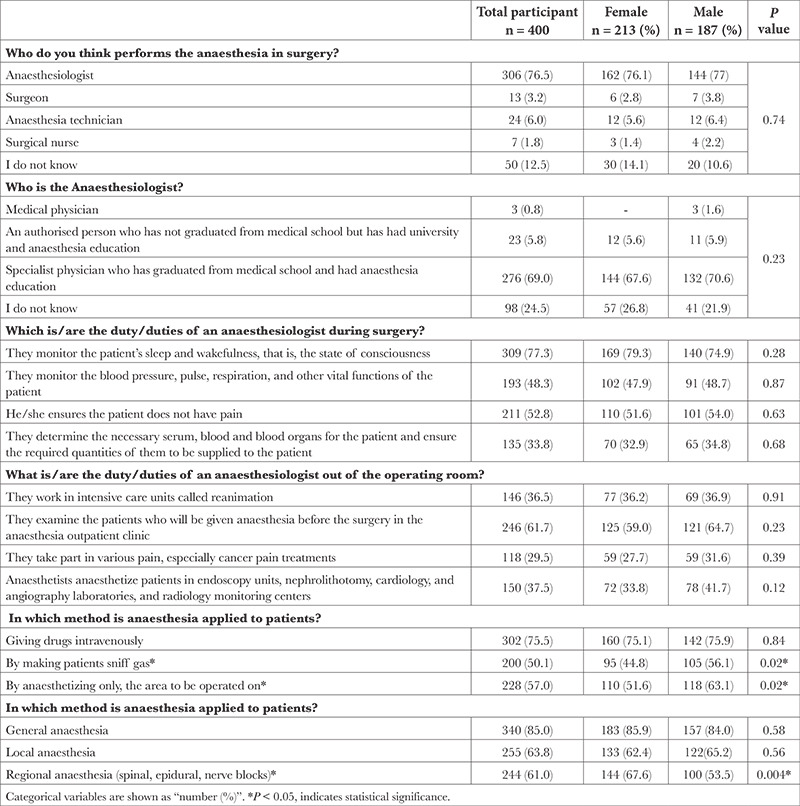
Knowledge Level of Patients/Patient Relatives About Anaesthesiologists and Anaesthesia Practices

**Table 3:**
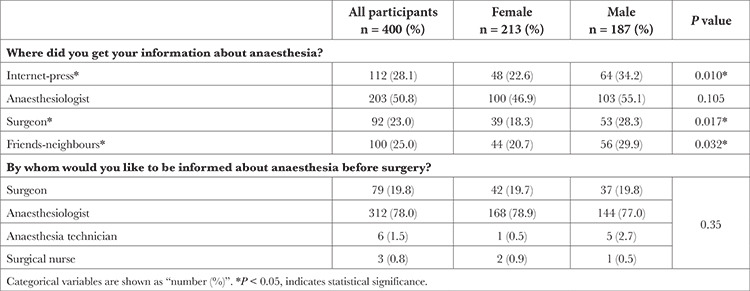
Methods used by Patients/Patient Relatives for Obtaining Information About Anaesthesia

**Table 4:**
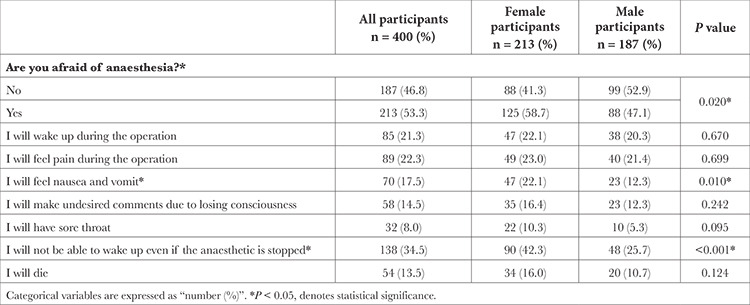
Participant Patients’/Patient Relatives’ Fears About Anaesthesia
